# GABA_A_ receptors as plausible molecular targets and mediators for taurine and homotaurine actions

**DOI:** 10.3389/fphar.2023.1271203

**Published:** 2023-12-14

**Authors:** Pratap Meera, Mikko Uusi-Oukari, Gerald S. Lipshutz, Martin Wallner

**Affiliations:** ^1^ Department of Neurobiology, University of California, Los Angeles, Los Angeles, CA, United States; ^2^ Integrative Physiology and Pharmacology, Institute of Biomedicine, University of Turku, Turku, Finland; ^3^ Department of Surgery, University of California, Los Angeles, Los Angeles, CA, United States; ^4^ Molecular and Medical Pharmacology, University of California, Los Angeles, Los Angeles, CA, United States; ^5^ Intellectual and Developmental Disabilities Research Center, University of California, Los Angeles, Los Angeles, CA, United States; ^6^ Semel Institute for Neuroscience, University of California, Los Angeles, Los Angeles, CA, United States

**Keywords:** taurine, homotaurine, inflammation, GABA_A_ receptor, Alzheimer’s disease, GABA-mimetics, tramiprosate

## Abstract

Dementia and autoimmune diseases are prevalent conditions with limited treatment options. Taurine and homotaurine (HT) are naturally occurring sulfonate amino acids, with taurine being highly abundant in animal tissues, but declining with age in the blood. HT is a blood-brain barrier permeable drug under investigation for Alzheimer’s disease. HT also has beneficial effects in a mouse model of multiple sclerosis likely through an anti-inflammatory mechanism mediated by GABA_A_ receptor (GABA_A_R) agonism in immune cells. While both taurine and HT are structural GABA analogs and thought to be GABA mimetics at GABA_A_Rs, there is uncertainty concerning their potency as GABA mimetics on native GABA_A_Rs. We show that HT is a very potent GABA mimetic, as it evokes GABA_A_R-mediated currents with an EC_50_ of 0.4 μM (vs. 3.7 μM for GABA and 116 µM for taurine) in murine cerebellar granule cells in brain slices, with both taurine and HT having similar efficacy in activating native GABA_A_Rs. Furthermore, HT displaces the high affinity GABA_A_R ligand [^3^H]muscimol at similarly low concentrations (HT IC_50_ of 0.16 μM vs. 125 μM for taurine) in mouse brain homogenates. The potency of taurine and HT as GABA_A_R agonists aligns with endogenous concentrations of taurine in the blood and with HT concentrations achieved in the brain following oral administration of HT or the HT pro-drug ALZ-801. Consequently, we discuss that GABA_A_Rs subtypes, similar to the ones we studied here in neurons, are plausible targets for mediating the potential beneficial effects of taurine in health and life-span extension and the beneficial HT effects in dementia and autoimmune conditions.

## Introduction

While GABA is the main inhibitory neurotransmitter in the mammalian brain with actions primarily mediated by ionotropic GABA_A_ receptors (GABA_A_Rs), there is growing evidence of pharmacologically similar GABA_A_Rs expressed in immune cells (Bhandage and Barragan, 2021; Kang et al., 2022; Tian and Kaufman, 2023). Activation of these immune cell GABA_A_Rs could provide a plausible explanation why classical GABA_A_Rs agonists (alcohol, barbiturates and anesthetics like etomidate and propofol) exert anti-inflammatory, immunosuppressive actions (Wheeler et al., 2011; Prud’homme et al., 2015; Szabo and Saha, 2015), and also why moderate alcohol consumption is protective against autoimmune disease (Azizov et al., 2020).

Taurine is a highly abundant non-proteinogenic amino acid with a multitude of proposed biological functions (Huxtable, 1992; Warskulat et al., 2007), including agonist actions on GABA_A_ and glycine receptors (Albrecht and Schousboe, 2005; Jia et al., 2008). Taurine levels in the blood decline with age, and it has been shown that high doses of taurine (1 g/kg per day) in mice increases life span by ∼10% and also had beneficial effects on health-span, suggesting a potential role of taurine in aging, with an unclear mechanism of action (Singh et al., 2023).

**FIGURE 1:**
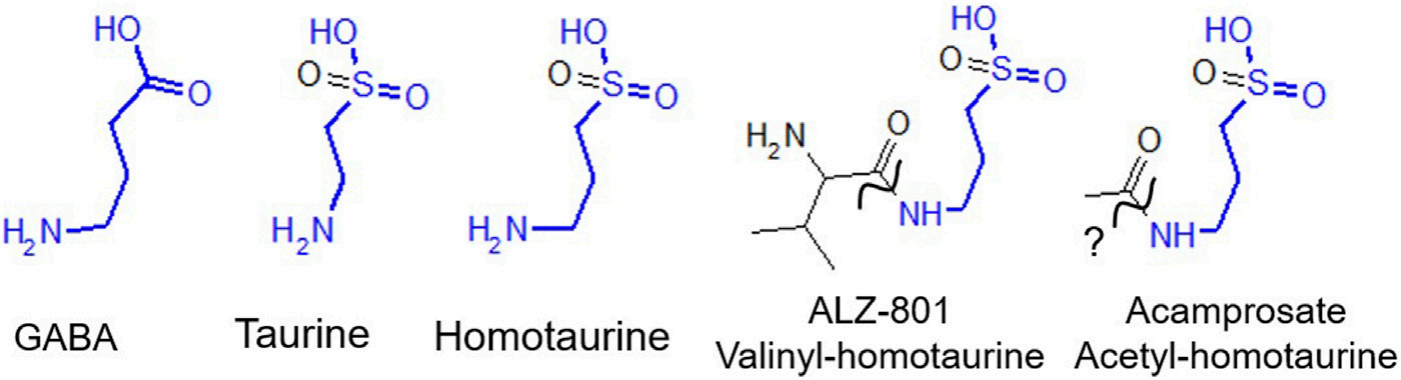
Homotaurine and taurine are structural GABA analogs with close structural similarities with GABA, as indicated in blue. GABA_A_ receptors are the main inhibitory neurotransmitter in the mammalian brain; however, they are also expressed on many immune cells. Taurine is highly abundant in mammals and was recently implicated as a driver of aging. Homotaurine (a.k.a. Alzhemed or Tramiprosate) was in clinical trials for Alzheimer’s disease (AD). ALZ-801 (Valiltramiprosate) is valine-conjugated homotaurine and an oral pro-drug, hydrolyzed into valine and homotaurine by amidase (squiggly line) and in clinical trials for AD in high-risk APOE4 homozygous patients showing cognitive decline. Acamprosate is N-acetyl-homotaurine and FDA approved since 2004 to treat alcohol abuse and generally thought not to be converted into HT and acetate (squiggly line with question mark).

Homotaurine (HT) is structurally closely related to taurine having an additional carbon in its backbone (Figure 1). While abundant in, e.g., seaweed (Terriente-Palacios and Castellari, 2022), HT has not been reported in vertebrates. Based on the ability of 100 μM HT to reduce β-amyloid (Aβ) toxicity in primary cultured neurons and reduce amyloid plaques in a murine model (Gervais et al., 2007), and evidence from *in vitro* studies that HT, by binding to soluble Aβ, reduces oligomer formation and aggregation (Kocis et al., 2017), HT (Alzhemed/Tramiprosate) was developed as a potential treatment for AD. While HT did not meet the expectations in clinical trials for slowing cognitive decline, it showed cognitive improvement in APOE4 carriers and an excellent safety profile (Manzano et al., 2020). A pro-drug version of HT, valine-conjugated HT (ALZ-801 or Valiltramiprosate) was designed to be better tolerated and a more effective, longer lasting form of HT. Orally administered ALZ-801 is efficiently metabolized by endogenous amidases into valine and the active compound HT in humans (Hey et al., 2018). ALZ-801 is currently in a Phase 3 clinical trial in APOE4/4 homozygous AD patients (Clinical Trials Identifier NCT04770220).

In the context of ALZ-801 being a pro-drug it is interesting to note that acetyl-homotaurine (Acamprosate, see Figure 1) is an FDA approved drug to treat alcohol cravings. Interestingly, both acetyl-homotaurine and HT have been shown to reduce alcohol intake in rats likely by interfering with dopamine release in the mesolimbic reward system (Olive et al., 2002). Unlike the structurally closely related ALZ-801 (valinyl-homotaurine), acamprosate (acetyl-homotaurine) (see Figure 1) has been reported (in a review) not to be metabolized, although details and data leading to this conclusion are not publicly available (Saivin et al., 1998).

A unique characteristic of HT is its ability to pass through the blood-brain barrier (BBB), unlike most other hydrophilic molecules, such as GABA (Gervais et al., 2007; Hey et al., 2018). Given HT’s hydrophilicity, it seems likely that BBB permeation occurs via an active transport mechanism, possibly utilizing the ubiquitously expressed (including the BBB) taurine transporter (Tsuji and Tamai, 1996; Qian et al., 2000). HT’s ability to pass through the BBB is the likely explanation for why HT, but not GABA, effectively alleviated experimental brain autoimmune encephalomyelitis (EAE) in a murine model of multiple sclerosis (Tian et al., 2018; Tian et al., 2021). HT’s disease-modifying abilities are likely mediated through GABA_A_Rs on immune cells, as well as on GABA_A_Rs on brain microglia, with GABA_A_R activation exerting anti-inflammatory actions on immune cells (Lee et al., 2011; Bhandage and Barragan, 2021; Tian and Kaufman, 2023). Other non-BBB-permeable GABA_A_R ligands, like GABA itself, have been shown to ameliorate peripheral autoimmune diseases, such as type I diabetes and rheumatoid arthritis, as well as inflammation stemming from type II diabetes and SARS-CoV-2-infection (COVID-19) in animal models [reviewed in Tian and Kaufman (2023)].

Both taurine and HT are structural GABA analogs, the only difference being the substitution of the carboxyl group of GABA with a sulfonate group for HT (Figure 1). Taurine has been reported to displace [^3^H]muscimol with an average IC_50_ of ∼50 μM from rat brain GABA_A_Rs (Bureau and Olsen, 1991). At concentrations of ≥10 μM taurine has been shown to activate GABA_A_Rs in thalamocortical relay neurons and at concentrations >100 μM, taurine also activates strychnine-sensitive glycine receptors (Jia et al., 2008). HT has also long been thought to be a GABA agonist since it stimulates GABA_A_R-mediated ^36^Cl^−^ chloride flux, albeit only at fairly high concentrations of ≥100 μM (Allan and Harris, 1986). In both cultured murine astrocytes and the STC-1 cell line, with features of native intestinal enteroendocrine cells, both homotaurine and taurine evoke GABA_A_R-mediated currents at high (1 mM) concentrations (Reyes-Haro et al., 2014).

We show that both HT and taurine are GABA_A_R mimetics with HT about 10 times more potent than GABA itself and more than 100 times more potent when compared to taurine. HT’s BBB permeability and its potent GABA_A_R agonist activity makes it an excellent candidate to exert anti-inflammatory effects on GABA_A_Rs expressed on immune cells, microglia, and astrocytes in CNS disorders involving inflammation, such as MS and AD (Lee et al., 2011; Kang et al., 2022; Jorfi et al., 2023; Tian and Kaufman, 2023). Additionally, considering the reported life-span prolonging effects of (massive) taurine supplementation (Singh et al., 2023), with immune cell GABA_A_Rs as a likely target, the data presented here that HT is about 100 times more potent than taurine suggest that HT, or a pro-drug alternative like vali-tramiprosate should be tested for beneficial effects on life- and health-span. It will be important for future work to elucidate details how GABA and GABAergic drugs can modulate the activity of GABA_A_R subtypes expressed in different types of immune cells and how this impacts immune function.

## Methods

### Animal procedures

Many procedures were described recently (Meera et al., 2023). Animal studies were performed according to protocols approved by the University of California at Los Angeles (UCLA) Chancellor’s Animal Research Committee (Animal Welfare Assurance number: ARC-2019-032). Wild-type (C57BL/6, WT) mice (age 1.5–8 months of age, both sexes, total ∼30 mice) were used for the studies. The mice were briefly anesthetized, killed by decapitation and brains removed. The cerebellum of 6–14 weeks old mice was used to prepare slices for electrophysiology. For ligand binding studies brains were stored at −80°C and shipped on dry ice.

### Reagents

[Methylene-^3^H]muscimol (22 Ci/mmol) was purchased from PerkinElmer Life and Analytical Sciences (Boston, MA, United States). Taurine (T0625), homotaurine (A76109) and GABA (A2129) were purchased from Sigma-Aldrich (St. Louis, MO, United States) and prepared as 10 mM stock solutions either in artificial cerebrospinal fluid (aCSF, electrophysiology) or in assay buffer for [^3^H]muscimol displacement studies.

DNQX, TTX and Gabazine (SR95531) were purchased from Tocris Cookson, Inc. (Part of Bio-Techne, Minneapolis, MN, United States) or Hello-Bio (Princeton, NJ, United States).

### Brain slice preparation and electrophysiology

Cerebellar slices were prepared using standard techniques (Meera et al., 2011). A vibratome (Leica VT-1000s, Deer Park, IL, United States) was used to prepare 285–300 µm thick slices. The artificial cerebrospinal fluid (aCSF) for electrophysiological recordings and storage was saturated with 95% O_2_ and 5% CO_2_ and consisted of (in mM): 119 NaCl, 26 NaHCO_3_, 11 glucose, 2.5 KCl, 2.5 CaCl_2_, 1.3 MgCl_2_, and 1 NaH_2_PO_4_ (chemicals from Sigma-Aldrich, St Louis, MO, United States). The whole cell pipette solution consisted of (in mM): 100 KCl, 5 NaCl, 40 HEPES, 4 MgCl_2_, 4 ATP and 0.4 GTP, titrated to pH 7.4 with KOH. Cerebellar granule cells (CGCs) were visualized using an upright microscope (Zeiss, White Plains, NY, United States) equipped with infrared-DIC enhancement. Pipettes with resistances between 10–12 MΩ and a Multiclamp 700B amplifier (Axon Instruments, Inc., Foster City, CA United States) were used to record from CGCs. Recordings were digitized at 10 kHz and filtered at 4 kHz. Neurons were voltage-clamped at −70 mV at room temperature with 10 µM DNQX (to block glutamate receptors) and action potentials blocked by 0.3 µM TTX.

### Preparation of brain membranes

Adult mouse brain (*sans* cerebellum) membrane pellets were prepared essentially as described by Uusi-Oukari et al. (2014), suspended in 50 mM Tris-HCl, pH 7.4 (assay buffer) and frozen at −80°C. For binding experiments, the suspension was thawed and washed by centrifugation/resuspension in assay buffer.

5 nM [^3^H]muscimol binding was measured in assay buffer at room temperature (RT, ∼22°C) in a total volume of 300 µL. 100 μM GABA was added to determine non-specific binding. After 20 min incubation at room temperature the membranes were collected with a Brandel Cell Harvester (Model M-24, Gaithersburg, MD, United States) onto Whatman GF/B filters (Whatman International Ltd., Maidstone, United Kingdom). The samples were rinsed twice with 4–5 mL of ice-cold assay buffer. Filtration and rinsing steps took a total time of ∼15 s. Air-dried filters were immersed in 3 mL of Optiphase HiSafe 3 scintillation fluid (Wallac, Turku, Finland) and radioactivity determined in a Hidex 600 SL liquid scintillation counter (Hidex, Turku, Finland).

### Data analysis

[^3^H]Muscimol displacement and electrophysiology data were analyzed with GraphPad Prism 7 software (GraphPad, San Diego, CA, United States). Data are presented as mean ± standard error of mean (SEM). Statistical data analysis was performed with a Student’s t-test (two-tailed distribution, unequal variance) in Microsoft Excel.

## Results

Given the therapeutic applications of the GABA structural analogs HT and taurine as potential drugs and supplements, we conducted whole-cell electrophysiology on cerebellar granule cells (CGCs) in mouse brain slices to study the potency of HT and taurine on native GABA_A_Rs. We decided to record GABA_A_Rs in CGC neurons since they are the most numerous neurons (>50%) in the mammalian brain (Consalez et al., 2021) and express both abundant synaptic as well as highly GABA-sensitive extrasynaptic δ subunit-containing GABA_A_Rs (Farrant and Nusser, 2005). In addition, we used the high affinity GABA_A_R-specific ligand [^3^H]muscimol to study its displacement by taurine and HT in total mouse brain (excluding the cerebellum) homogenates to ensure that: 1) HT and taurine GABA mimetic effects are due to GABA-mimetic actions on the orthosteric GABA-site on GABA_A_Rs and 2) that any electrophysiological effects are not specific for the somewhat unique GABA_A_Rs subtypes expressed in cerebellar granule cells.

To mimic the effect of the expected gradual changes in extracellular HT and taurine concentrations in the brain, either after peripheral/oral application (HT) or by potential non-synaptic release (in case of taurine under hyperexcitability conditions), we recorded cumulative concentration-response curves using taurine, HT and GABA. Under these conditions, i.e., in the continuous presence of agonists, it seem likely that abundant synaptic receptors are to a large extent desensitized and therefore it seems plausible that HT, taurine and GABA evoked currents should be at least partly carried by extrasynaptic GABA_A_Rs (mostly composed of fairly abundant α6β3δ subunit GABA_A_Rs in CGCs) which show not only higher GABA affinity but also much slower desensitization, both necessary to mediate a constant (tonic) form of inhibitory GABA_A_R currents (Wallner et al., 2003; Farrant and Nusser, 2005; Meera et al., 2011).

**FIGURE 2:**
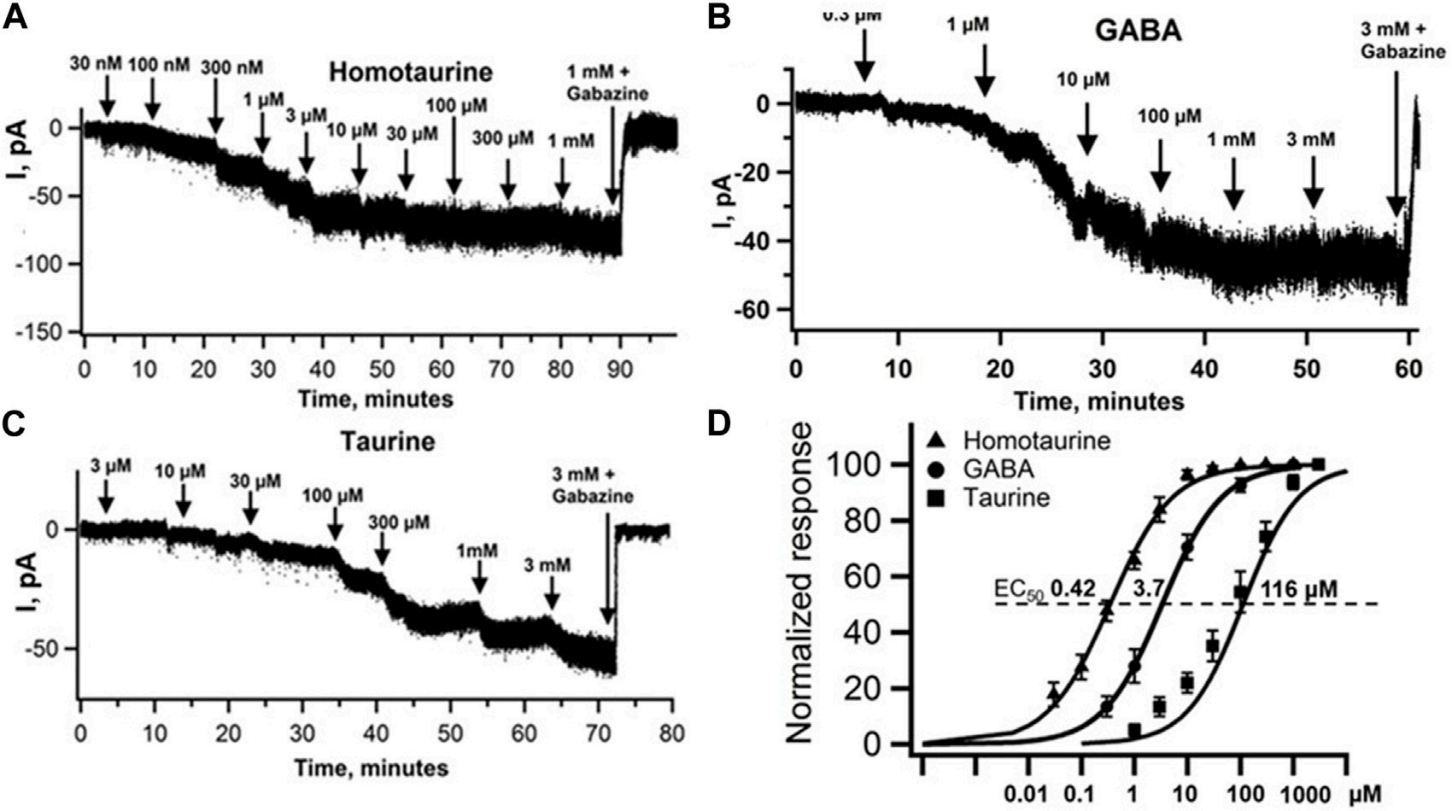
Homotaurine and Taurine are GABA mimetics evoking tonic GABA_A_R currents in mouse cerebellar granule cells (CGC). Cumulative HT **(A)**, GABA **(B)** and taurine **(C)** concentration-response curves using whole-cell patch clamp recordings of CGCs held at −70 mV in the presence of the glutamate receptor blocker DNQX (dinitroquinoxalinedione, 10 μM) and action potentials blocked by 0.3 μM TTX (tetrodotoxin). After application of 1 mM HT, and 3 mM GABA or taurine, the tonic GABA current was blocked by the GABA_A_R specific blocker Gabazine (SR95531). **(D)** Normalized concentration-response curves of HT (▲) with an average EC_50_ ± SEM value of 425 ± 7 nM, Hill slope 0.79 ± 0.05 (*n* = 7), GABA (●) EC_50_ = 3.7 ± 0.5 μM, Hill slope 0.92 ± 0.13 μM (*n* = 7) for taurine (■) 116 ± 13 μM, Hill Slope 0.87 ± 0.06 (*n* = 7). Statistical analysis with Student’s t-test (two-tailed distribution, two-sample unequal variance) shows that the differences in EC_50_ values between HT, GABA and taurine are highly significant: t-test EC_50_ comparison between GABA and HT is *p* = 0.0003, and GABA vs. taurine *p* = 0.0001. Taurine vs. HT *p* = 0.0001, whereas the Hill slopes of the HT, taurine and GABA curves are not different (all *p* > 0.32). Maximal current responses with 1 mM HT were (I_max_±SEM) −75 ± 7 pA (*n* = 7), 3 mM taurine −67 ± 7 pA and 3 mM GABA −65 ± 5 pA (*n* = 7) with *p* values for I_max_ GABA vs. HT *p* = 0.35, GABA vs. taurine *p* = 0.87 and HT vs. taurine *p* = 0.47.

HT elicited currents with a threshold concentration of ∼100 nM under our recording conditions, reaching saturation at around 10 µM (Figure 2A). On the other hand, taurine exhibited threshold concentrations of around 10 μM and reached saturation only at around 3 mM (Figure 2C). The half maximal concentration (EC_50_) for evoking current was 0.425 ± 0.007 µM (n = 7) for HT (Figure 2D and 116 ± 13 µM (*n* = 7) for taurine (Figure 2D). We also used GABA under the same recording conditions and show that sequential application of increasing GABA concentrations elicited currents with threshold activation concentration of 300 nM with currents saturating at ∼1 mM, with a GABA EC_50_ of 3.7 ± 0.5 μM (*n* = 7) (Figure 2D). Statistical analysis using Student’s t-test showed highly significant differences in potency (EC_50_) between taurine, GABA and HT with *p*-values: GABA vs. taurine *p* = 0.0001, GABA vs. HT *p* = 0.0025 and taurine vs. HT *p* = 0.0001.

To assess to what extent HT, GABA and taurine evoked currents are mediated by GABA_A_Rs, we applied Gabazine (a.k.a. SR95531) a specific GABA_A_R antagonist together with a saturating concentration of 3 mM taurine, 1 mM HT or 3 mM GABA. Maximal currents were −75 ± 7 pA for HT (n = 7), −67 ± 7 pA for taurine (*n* = 7) and −66 ± 6 pA for GABA (*n* = 7). No significant differences in maximal current amplitudes were seen using Student’s t-test: GABA vs. taurine *p* = 0.87, GABA vs. HT *p* = 0.35 and taurine vs. HT *p* = 0.47. As depicted in Figures 2A–C, application of Gabazine completely blocked currents evoked by 1 mM HT, 3 mM taurine and 3 mM GABA.

**FIGURE 3:**
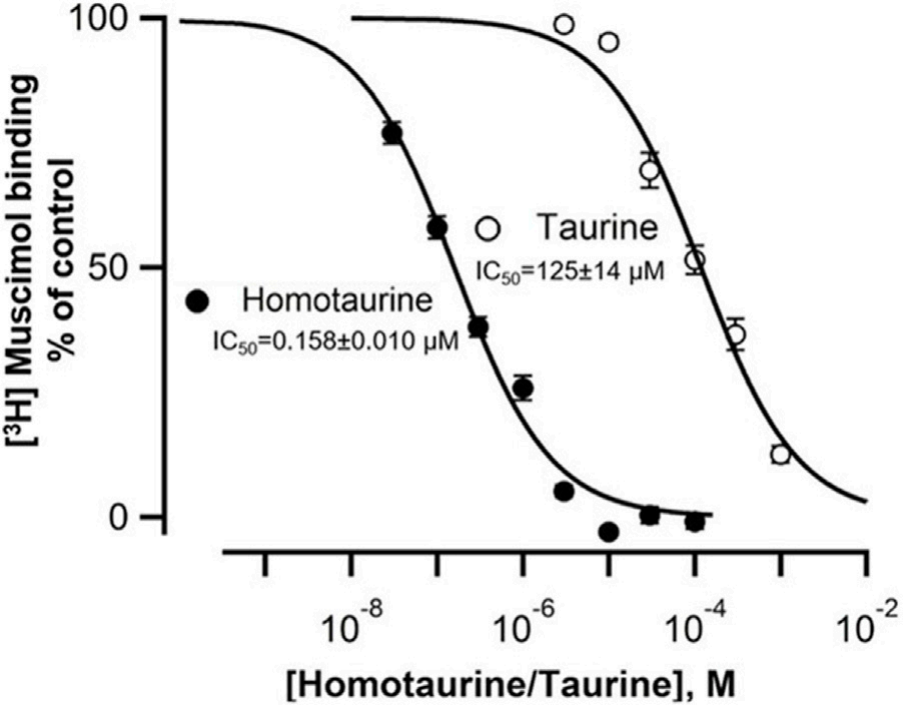
Homotaurine and Taurine displacement of 5 nM [^3^H]muscimol from mouse brain native GABA_A_Rs identifies HT as a high affinity GABA-site ligand. HT and taurine displace the high affinity GABA_A_R GABA-site ligand [^3^H]muscimol in a concentration-dependent manner. The half maximal inhibitory concentration (IC_50_) values (mean ± SEM) are for HT IC_50_ = 158 ± 10 nM, Hill slope −0.79 ± 0.04 (*n* = 9); for taurine IC_50_ = 125 ± 14 μM, Hill slope −0.86 ± 0.04 (determined from n = 9 displacement curves, from three mouse brains *sans* cerebellum). Statistical comparison using a Student’s t-test (two-tailed distribution, unequal variance) shows that the HT and taurine IC_50_ values are significantly different (*p* = 0.000018) whereas the Hill slopes show no significant difference (*p* = 0.21).

Considering that cerebellar granule cell GABA_A_Rs are somewhat unique due to the expression of the GABA_A_R α6 subunit, we tested the potency of both taurine and HT in total mouse brain (excluding the cerebellum) using the high affinity GABA_A_R orthosteric radioligand [^3^H]muscimol at room temperature. Figure 3 shows that HT displaces [^3^H]muscimol binding in mouse brain membranes with a half maximum inhibitory concentration (IC_50_) of 0.158 ± 0.010 µM (*n* = 9) for HT and 125 ± 14 µM (*n* = 9) for taurine.

## Discussion

We show here that HT is a surprisingly potent GABA_A_R agonist with an EC_50_ for activating native currents in cerebellar granule cells of ∼0.4 µM and an IC_50_ for displacing the high affinity GABA_A_R site ligand [^3^H]muscimol of 0.16 µM (Figures 2, 3). While HT has long been suggested to be a GABA_A_R agonist, e.g., by showing that ≥100 µM HT can evoke GABA_A_R mediated ^35^Cl^−^-flux in synaptosomes (Allan and Harris, 1986), to our knowledge this is the first demonstration that HT is a highly potent GABA_A_R agonist. In addition, our data are consistent with studies that HT, taurine and GABA suppress the firing frequency in a concentration-dependent manner in guinea pig cerebellar Purkinje cells (Okamoto and Sakai, 1981). Our findings reported here suggest that this suppression of Purkinje firing by HT and taurine is primarily mediated by GABA_A_R activation. This conclusion is consistent with our findings that the GABA_A_R specific antagonist Gabazine completely blocks taurine-evoked currents in cerebellar granule cells, suggesting that glycine receptor activation at up to 1 mM HT and 3 mM taurine is negligible in adult murine CGCs (Figures 2A, C). This is consistent with the reported small glycine-evoked currents in GCGs in very young (1 week old) rats, which essentially disappear during maturation (Kaneda et al., 1995). This contrasts with findings in thalamic neurons in adult murine brain slices, where low taurine concentrations (50 μM) preferentially activate GABA_A_Rs, while higher (500 μM) taurine concentrations also evoke strychnine-sensitive glycine receptor currents (Jia et al., 2008).

Homotaurine and taurine have been reported to activate picrotoxin-sensitive GABA_A_R currents in cultured astrocytes and the STC-1 cell line. However, only high concentrations of HT (1 mM for astrocytes and 30 mM for the STC-1 cell line) and taurine (1 mM in both cell types) were tested (Reyes-Haro et al., 2014). Recombinant expression of homo-pentameric ρ1-GABA_A_Rs (formerly classified as GABA_C_ receptors) in oocytes produced HT-sensitive GABA_A_Rs with an EC_50_ of ∼300 μM, and it was shown that even saturating HT concentrations produced currents with amplitudes much smaller than those evoked by saturating GABA, and also that HT applied together with GABA led to reduction in evoked currents, all indicating that HT is a partial agonist on recombinantly expressed ρ1-GABA_A_Rs and therefore could act as a functional GABA_A_R antagonist (Ochoa-de la Paz et al., 2018). We report here that native GABA_A_R in CGCs are about 1000 times more sensitive to HT and show similar maximal currents at saturating ligand concentrations, indicating that GABA, taurine and HT at saturating concentrations have similar propensity to open GABA_A_R Cl^−^ channels under our experimental conditions. Low ligand efficacy for GABA itself has been widely reported with recombinant expression of extrasynaptic GABA_A_Rs, which show high GABA sensitivity and carry the δ subunit in place of the γ2 subunit (Bianchi and Macdonald, 2003; Meera et al., 2009). Also, the GABA-mimetic Gaboxadol (a.k.a. THIP) has not only higher potency but also higher efficacy than GABA itself in recombinantly expressed extrasynaptic types of GABA_A_Rs lacking the γ2 subunit (Bianchi and Macdonald, 2003; Meera et al., 2011). Given that it is likely that currents that we record here are at least partially mediated by high affinity extrasynaptic receptor subtypes (as mentioned above), we would like to note that it is possible that GABA itself is a partial agonist and all we can conclude is that HT, taurine and GABA all have similar efficacy under our recording conditions.

While the overall taurine concentration in the mammalian brain is very high (3–9 mM range), most of this taurine is contained intracellularly with a large concentration gradient across the plasma membrane and limited extracellular taurine in the resting state, ranging from 1–10 µM (Lerma et al., 1986; Caine and Geracioti, 2016). However, during neuronal depolarization (e.g., in pronounced hyper-excitability states like seizures), the local extracellular taurine concentration can reach ∼120 µM (Albrecht and Schousboe, 2005), a concentration right in the range (see Figures 2, 3) where it might activate GABA_A_Rs. Therefore taurine release under neuronal hyper-excitability conditions could provide an opposing inhibitory influence by activating classical neuronal GABA_A_Rs (Oja and Saransaari, 2013). Taurine concentrations in human blood (serum) have been found to range between ∼250 and 30 µM with the highest levels at very young ages, markedly decrease with age with taurine supplementation increasing life-span by 10% in mice (Singh et al., 2023). Again, the potency of taurine we report here to activate GABA_A_Rs is right in the range to mediate such effects on aging, possibly through anti-inflammatory actions mediated by GABA_A_Rs expressed on immune cells.

In NIH-Swiss wild type mice, plasma taurine is around 500 μM and more than doubles in arginase-deficient mice (Cantero et al., 2016). The reason behind this elevation in blood taurine in murine arginase deficiency is not understood. One possibility is that taurine somehow counteracts the toxicity of dramatically increased plasma ammonia. Another, not mutually exclusive, possibility is that increased taurine is simply a consequence of cellular toxicity (likely caused by hyperammonemia) with increased release of intracellular taurine from damaged cells.

Protective effects of taurine and HT against Aβ-induced neurotoxicity have been reported; in chick embryonic neuronal cultures, 1 mM taurine prevented the neurotoxicity of 44 μM Aβ_42_ and glutamate receptor agonists. The protective effect of taurine in cultured neurons was blocked by the GABA_A_R blocker picrotoxin, suggesting that taurine’s neuroprotection was mediated by GABA_A_R activation (Louzada et al., 2004). Similarly, 100 μM HT (Tramiprosate, Alzhemed) inhibited neuronal death in cultures induced by 5 μM Aβ_42_ (Gervais et al., 2007). These findings suggest that taurine and HT can counteract possible direct excitotoxic effects of soluble Aβ through GABA_A_R activation. In addition, it was reported that HT prevented Aβ aggregation and amyloid formation; it was observed that 100 μM HT prevented a structural shift of Aβ_40_ from random coil to β-sheet (Gervais et al., 2007). Furthermore, in a solution containing 22 μM Aβ_42_, the addition of increasing concentrations of HT (from 220 to 22,000 μM) resulted in the association of up to 6 HT molecules “enveloping” a single homomeric Aβ42 peptide to prevent aggregation (Kocis et al., 2017). It is important to note that the concentrations of HT that prevented Aβ aggregation *in vitro* are orders of magnitude higher than the estimated average total brain ^14^C-HT of 0.55 μM (Hey et al., 2018). In marked contrast to the effects of ≥100 μM HT on amyloid formation described above, a concentration of 0.5 μM HT nicely aligns with the potency of HT on GABA_A_Rs reported here (EC_50_∼0.4 μM for GABA_A_R activation, IC_50_∼0.2 μM for [^3^H]muscimol displacement). Accordingly, the main beneficial effects of HT and taurine could be mediated by GABA_A_Rs that limit the inflammatory actions of microglia, astrocytes, and CNS-infiltrating immune cells. This is consistent with “Inflammaging,” a term coined describing the concept of inflammation as a critical factor in the aging process (Franceschi et al., 2018).

Our finding here that HT is a highly potent GABA agonist, and the report that ALZ-801 is a HT pro-drug, suggests that revisiting the issue whether acetyl-homotaurine (Acamprosate) is also metabolized and a HT pro-drug, should be considered particularly considering a report that HT at ∼10 fold lower doses than acetyl-HT reduces alcohol intake in rat models and alcohol-induced dopamine release in the nucleus accumbens (Olive et al., 2002); A likely mechanism how taurine and HT suppress the immune system is through the activation of GABA_A_Rs expressed on CNS-infiltrating T cells and macrophages (Kang et al., 2022; Prud’homme et al., 2015; Tian and Kaufman, 2023), and/or also CNS microglia mediating anti-inflammatory functions in the brain (Lee et al., 2011; Jorfi et al., 2023). The activation of such non-neuronal GABA_A_Rs on immune cells may also provide a plausible explanation why essentially all GABA_A_R agonists (i.e., barbiturates, etomidate, propofol, benzodiazepines, alcohol, etc.) suppress immunity (Wheeler et al., 2011; Prud’homme et al., 2015; Szabo and Saha, 2015) and such immune-modulatory anti-inflammatory actions could explain the putative beneficial effects of moderate alcohol consumption on health and longevity (Klatsky, 2003). An alternative, not mutually exclusive, possibility for the beneficial effects of HT and taurine is their ability to counteract potential Aβ-mediated neuronal excitotoxicity through GABA_A_R activation (Louzada et al., 2004; Gervais et al., 2007).

The potential anti-inflammatory actions of taurine and HT mediated by GABA_A_Rs are consistent with evidence implicating inflammation in the development of AD. It is thought that amyloid plaques attract immune cells, leading to inflammation that results in neuronal damage, cognitive deficits and brain atrophy, which are characteristic of advanced AD (Heppner et al., 2015; Jorfi et al., 2023). This model provides a plausible explanation for the observation that although amyloidosis is a hallmark feature of AD, not all individuals with significant amyloid deposition exhibit cognitive decline or progress to develop AD, presumably because they lack an inflammatory neurotoxic immune response. This phenomenon is referred to as NDAN (**n**on-**d**emented with **A**D **n**europathology) (Kok et al., 2022). There is compelling evidence linking inflammation not only to AD but also to other devastating neurodegenerative disorders [such as Parkinson’s disease, amyotrophic lateral sclerosis and multiple sclerosis (Glass et al., 2010)], and with taurine as a driver of aging (with GABA_A_Rs as likely targets), this may include aging in general (Singh et al., 2023).

In conclusion, HT and taurine, as GABA_A_R agonists, highlight their potential as promising anti-inflammatory agents with conceivably broad implications for various diseases. This includes neurodegenerative disorders such as AD and autoimmune diseases, exemplified by the beneficial effects of HT in AD and on a mouse model of multiple sclerosis. Harnessing the anti-inflammatory properties of taurine and HT could open new avenues for developing interventions aimed at extending both lifespan and health span, ultimately improving overall well-being and quality of life.

## Data Availability

The raw data supporting the conclusion of this article will be made available by the authors, without undue reservation.

## Abbreviations

AD, Alzheimer’s disease; Aβ, Amyloid β-peptide; BBB, Blood-brain barrier; CGC, Cerebellar granule cell neurons; CNS, Central nervous system; EAE, Experimental autoimmune encephalomyelitis; EC_50_, Half maximal effective concentration; GABA_A_R, γ-aminobutyric acid type A receptor; HT, Homotaurine, Tramiprosate, Alzhemed; IC_50_, Half maximal inhibitory concentration; I_max_, Maximal current response at saturating ligand concentration.
